# 1845. Integrating Infectious Diseases and Primary Care Providers in a Syringe Service Program in Chicago to Improve Access to Care for People with Substance Use Disorder

**DOI:** 10.1093/ofid/ofad500.1673

**Published:** 2023-11-27

**Authors:** Ryan D Knodle, Lelia H Chaisson, Karen F Cotler, Michael Huyck, Sarah Messmer, Stockton Mayer

**Affiliations:** University of Illinois at Chicago, Chicago, Illinois; University of Illinois at Chicago, Chicago, Illinois; University of Illinois at Chicago, Chicago, Illinois; University of Illinois at Chicago, Chicago, Illinois; University of Illinois at Chicago, Chicago, Illinois; University of Illinois at Chicago, Chicago, Illinois

## Abstract

**Background:**

People with substance use disorders (SUD) are at elevated risk of infectious diseases (ID) but have low uptake of preventive and primary care (PC) due to structural, social, and behavioral barriers to care. Syringe service programs (SSP) are established, trusted sources of care; expanding health care provided through these programs may result in improved health for this population. We aimed to assess uptake of services from a pilot program in Chicago that offered PC, ID consultation, and harm reduction services.

**Methods:**

PC and ID providers were embedded in the SSP to provide routine PC and preventive health services to patients seeking harm reduction services referred by outreach workers with lived SUD experiences. For this analysis, we reviewed all clinical encounters occurring from 10/19/2018-12/31/2021. We assessed patient demographics and visit types and determined the number of encounters for those with and without SUD. We compared characteristics of encounters for individuals with and without SUD using Chi-squared tests and Wilcoxon rank sum tests.

**Results:**

During the study period, a total of 552 patients had 1720 clinical encounters (median 1 per patient, range 1-44). Overall, 390 (70.7%) patients were male, median age was 43 years (IQR 19-71), and 415 (75.2%) had public insurance (Table 1). A higher proportion of those who had a visit for SUD had public insurance compared to those without SUD (79.6% vs 67.2%, p=0.003).

Of the 1720 encounters, 1227 (71.3%) included care for active SUD, of which 402 (23.4%) addressed infections often associated with SUD (Table 2). Encounters for HIV and hepatitis C virus made up 20% of all encounters. PC services were rendered at 910 (52.9%) encounters and 625 (36.3%) encounters included services for both SUD and PC. Those without active SUD were more likely to have encounters for PC (61.7% vs 49.4%, p< 0.001) and infections (50.7% vs 27.1%, p< 0.001) than those with active SUD.

**Figure:**
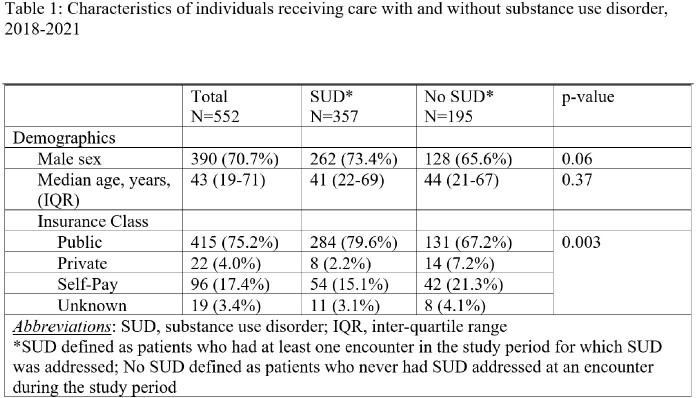

**Figure:**
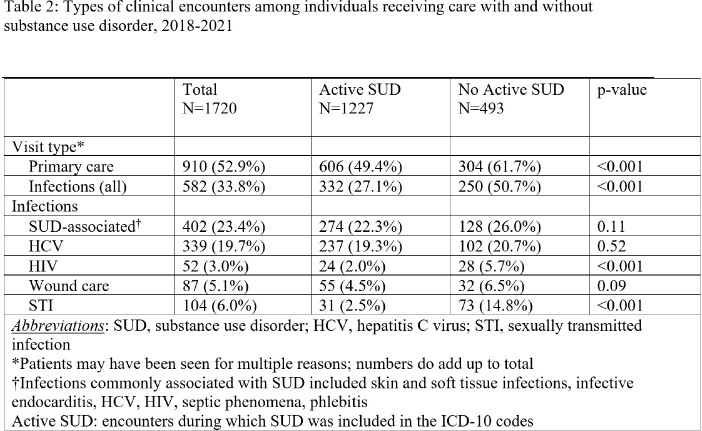

**Conclusion:**

Incorporating ID and PC services in an established SSP was effective for engaging vulnerable populations in Chicago, including people with SUD, with nearly half of all encounters among people with SUD including PC services. Integration of ID and PC within SSPs should be scaled-up to improve access to care, especially given the burden of infections in this population.

**Disclosures:**

**All Authors**: No reported disclosures

